# Coronavirus infections: Epidemiological, clinical and immunological features and hypotheses

**DOI:** 10.15698/cst2020.04.216

**Published:** 2020-03-02

**Authors:** Didier Raoult, Alimuddin Zumla, Franco Locatelli, Giuseppe Ippolito, Guido Kroemer

**Affiliations:** 1 Aix-Marseille Univ., Institut de Recherche pour le Développement (IRD), Assistance Publique - Hôpitaux de Marseille (AP-HM), MEPHI, 27 boulevard Jean Moulin, 13005 Marseille, France; IHU Méditerranée Infection, Marseille, France.; 2 Division of Infection and Immunity, Center for Clinical Microbiology, University College London, London, UK.; 3 The National Institute of Health Research Biomedical Research Centre at UCL Hospitals, London, UK.; 4 Department of Pediatric Hematology and Oncology IRCCS Ospedale Pediatrico Bambino Gesù, Rome, Italy.; 5 National Institute for Infectious Diseases, Lazzaro Spallanzani, IRCCS, Rome, Italy.; 6 Centre de Recherche des Cordeliers, Equipe labellisée par la Ligue contre le cancer, Université de Paris, Sorbonne Université, INSERM U1138, Institut Universitaire de France, Paris, France.; 7 Metabolomics and Cell Biology Platforms, Institut Gustave Roussy, Villejuif, France.; 8 Pôle de Biologie, Hôpital Européen Georges Pompidou, AP-HP, Paris, France.; 9 Suzhou Institute for Systems Medicine, Chinese Academy of Medical Sciences, Suzhou, China.; 10 Karolinska Institute, Department of Women's and Children's Health, Karolinska University Hospital, Stockholm, Sweden.

**Keywords:** Coronavirus, MERS-CoV, SARS-CoV, sARS-CoV-2, COVID-19, epidemiology, immunology

## Abstract

Coronaviruses (CoVs) are a large family of enveloped, positive-strand RNA viruses. Four human CoVs (HCoVs), the non-severe acute respiratory syndrome (SARS)-like HCoVs (namely HCoV 229E, NL63, OC43, and HKU1), are globally endemic and account for a substantial fraction of upper respiratory tract infections. Non-SARS-like CoV can occasionally produce severe diseases in frail subjects but do not cause any major (fatal) epidemics. In contrast, SARS like CoVs (namely SARS-CoV and Middle-East respiratory syndrome coronavirus, MERS-CoV) can cause intense short-lived fatal outbreaks. The current epidemic caused by the highly contagious SARS-CoV-2 and its rapid spread globally is of major concern. There is scanty knowledge on the actual pandemic potential of this new SARS-like virus. It might be speculated that SARS-CoV-2 epidemic is grossly underdiagnosed and that the infection is silently spreading across the globe with two consequences: (i) clusters of severe infections among frail subjects could haphazardly occur linked to unrecognized index cases; (ii) the current epidemic could naturally fall into a low-level endemic phase when a significant number of subjects will have developed immunity. Understanding the role of paucisymptomatic subjects and stratifying patients according to the risk of developing severe clinical presentations is pivotal for implementing reasonable measures to contain the infection and to reduce its mortality. Whilst the future evolution of this epidemic remains unpredictable, classic public health strategies must follow rational patterns. The emergence of yet another global epidemic underscores the permanent challenges that infectious diseases pose and underscores the need for global cooperation and preparedness, even during inter-epidemic periods.

## INTRODUCTION

Infections have been limiting quality of life and longevity throughout human history. Endemic infections have been associated to a significant number of avoidable deaths and a significant increase of morbidity all around the word. In addition, human-to-human transmission of newly emergent pathogens with pandemic potential regularly produce panic, with a negative impact on the economy and general welfare of large human communities.

The advent of public hygiene, vaccine and antibiotics has greatly reduced the probability to succumb to infectious disease and has improved the confidence of the public in the capacity of mitigating the possible consequences of infectious outbreaks. Nevertheless, also in recent times, several events produce significant concern in the general population. Panic spread as the consequence of overwhelming information on a series of potential epidemics rather than due to the actual diseases. These were the cases of Zika virus (a mild disease without any mortality excess whose impact is significant for vertical transmission only (increased rate of birth defect), [1, 2] variant Creutzfeldt–Jakob disease (“mad cow disease”) [3] and avian influenza [4]. The latter two diseases are both zoonotic infections without any pandemic potential due to the lack of efficient inter-human transmission. Moreover, the global community has successfully dealt with more scaring emerging agents. For example, the 2014-2015 Ebola hemorrhagic fever outbreak was successfully contained, and specific experience accrued during the West Africa epidemic, permitting to implement new vaccination strategies and therapeutic interventions Ebola outbreak (2014-2016) [5].

The study of respiratory viral infections has recently changed due to the standardized use of molecular biology in diagnostic tests. Therefore, molecular techniques today are not substantially influenced by the level of knowledge of those who practice them. Commercially available tests allow for the simultaneous detection of 20 common viral and bacterial pathogens within one hour, but tests leading to the identification of ever larger panels of infectious agents are under development [6].

## PATHOGENIC HUMAN CORONAVIRUSES

Among the causative agents of human respiratory tract infections are coronaviruses (CoVs) which are enveloped, single positive-strand RNA viruses belonging to the large subfamily Coronavirinae which infect birds and mammals. The viral RNA is the largest genome known and it is between 26 to 32 kilobases in length. There are seven CoVs known to cause human disease, which are divided into low pathogenic and highly pathogenic CoVs [7, 8]. Four coronaviruses (HCoVs, namely HCoV 229E, NL63, OC43, and HKU1), are known as non- severe acute respiratory syndrome (SARS)-like CoVs. They cause mild diseases and are globally endemic. Over the past two decades three highly pathogenic, novel zoonotic CoVs have emerged, which cause lethal human disease, and have thus generated much media hype and public concern: the SARS coronavirus (SARS-CoV now named SARS-CoV-1) discovered in November, 2002 [9], the Middle East respiratory syndrome (MERS) coronavirus (MERS-CoV) in June, 2012 [10]; and SARS-CoV-2, initially named 2019-nCoV when it was identified in December 2019 after sequencing of clinical samples from a cluster of patients with pneumonia in Wuhan, China [11]. The disease caused by SARS-CoV-2 is named Coronavirus Diseases-2019 (COVID-19)

All three CoVs are listed in the WHO Blueprint list for priority pathogens for research because of their epidemic potential and lack of effective treatments. SARS-CoV was first identified in humans in Guangdong, China, in November, 2002 and subsequently spread rapidly worldwide to 29 countries, resulting in 8098 human SARS cases with 774 deaths (9.6% mortality) [12]. The SARS epidemic ended abruptly in July, 2003, and no human cases of SARS have been detected since 2004. MERS-CoV was first isolated from a lung sample of an adult patient who died of pneumonia at the Al-Fakieh hospital in Jeddah, Saudi Arabia. MERS-CoV. A retrospective study then linked MERS-CoV to a hospital outbreak in April, 2012, in Jordan [13]. Dromedaries and camels are implicated as the zoonotic source of infection [14]. MERS-CoV continues to circulate and cause human disease with intermittent community clusters, and nosocomial outbreaks in the Middle East. MERS-CoV continues to cause human disease and as of Feb 29, 2020, there have been 2494 laboratory-confirmed human cases of MERS-CoV infection, with 858 deaths (34·0% mortality) reported from 27 countries in all continents, the majority of which were reported by Saudi Arabia (2106 cases, 780 deaths) [15].

## CLINICAL CHARACTERISTICS OF HUMAN CORONAVIRUS INFECTIONS

The clinical features of low pathogenic non-SARS CoV infections are undistinguishable from those found in patients with influenza virus (up to 61,000 lethal infections per year only in the US according to the Centers for Disease Control and Prevention, [16]). As with influenza and respiratory syncytial virus (RSV) infections, the majority of CoV infections usually lead to an asymptomatic or mild flu-like syndrome. Hence, without molecular diagnosis, these viral respiratory diseases, which all follow a seasonal pattern with a higher incidence in winter, are classed together as “flu”, irrespective of their exact infectious etiology. Non-SARS like CoV account for up to 20% of upper respiratory tract infections in adults [17] **(Table 1)**. However, non-SARS-like CoVs can be occasionally associated with severe acute respiratory illness (SARI) in the elderly, diabetics, and those with immunosuppression from any cause, although they have never been associated with major epidemics regionally or globally. In contrast, the highly pathogenic SARS-CoV and MERS-CoV have spurred locally intense short-lived fatal community and nosocomial outbreaks [18, 19]. SARS-like viruses are poorly adapted to the human host and unlike non-SARS-CoV viruses, they are generally associated with more severe clinical presentations.

**TABLE 1.** Respiratory tract infections with non-SARS coronaviruses.

Country	Number of cases investigated	Percentage positive	Reference
Brazil	775	7.6%	[65]
Brazil (slums)	282	21.2%	[66]
Hong Kong, China	4181	2.1%	[67]
Ghana	593	13.7%	[68]
Guangzou, China	13,048	2.3%	[69]
Guangzou, China	11,399	4.3%	[70]
Kenya	417	8.4%	[71]
Kenya	5,573	10.1%	[72]
South Africa	620 without TB	10.5%	[73]
	214 with TB	8%	
South Africa	860	4.8%	[74]
South Africa	1,026	15%	[75]
USA	854,575	4,6%	[76]

TB: tuberculosis.

The novel SARS coronavirus-2 (SARS-CoV-2) appears highly transmissible from human to human pathogen which causes a wide spectrum of clinical manifestations in patients with COVID-19 [19]. COVID-19 undoubtedly has attracted more attention by the mass news and social media than any other disease [20]. The rapid spread and ensuing public health control measures have led to unprecedented isolation and quarantine of large populations, international travel restrictions and economic paralysis of Wuhan, the entire Hubei province, vast areas of China followed by entire regions in South Korea and Northern Italy during January/February 2020. In these few weeks, there have been several scientific publications related to the epidemiology, clinical features, immunology and virological characteristics and a comparison of these could yield new insights into the pathogenesis of COVID-19.

## EPIDEMIOLOGY: RAPID SPREAD AND THE ROLE OF SYMPTOMATIC CASES

Despite seven years since first discovery, MERS-CoV transmission dynamics remain largely undefined and the roles of transmission, direct or indirect contact, airborne, droplet, or ingestion, have yet to be defined. MERS-CoV does not easily transmit from person to person unless contact with a MERS-CoV-infected subject is close. Several reports from Saudi Arabia describe transmission of silent or subclinical secondary infections after exposure to patients with MERS-CoV infections, some being apparently healthy individuals who were household contacts carrying MERS-CoV in their upper respiratory tract at low levels low amounts indicating that MERS-CoV can be transmitted from asymptomatic household or hospital contacts [21–23].

The ongoing SARS-CoV-2 outbreak has rapidly evolved and spread globally. As of Feb 29, 2020, there have been 83,652 laboratory confirmed cases of COVID-19, with 2791 deaths (3.4% mortality). Outside China, there have been 4691 cases reported from 51 countries with 67 deaths [24]. Whilst there have been several epidemiological links to bats, and other animals have been proposed, the source of primary SARS-CoV-2 transmission to humans remains unknown. The main clinical features of COVID-19 disease appear similar to other CoVs infections of humans. SARS-CoV-2 is generally associated with upper respiratory tract and high viral loads in upper respiratory tract secretions. This may evolve and progress towards SARI and in some cases to acute respiratory distress syndrome (ARDS), in the elderly, patients with comorbidities and immunosuppression [19]. Similar to other CoVs, it is now evident that health care services play an important role in the amplification of SARS-CoV-2 local epidemics. Asymptomatic carriage of SARS-CoV-2 has been described [25, 26] and the role of asymptomatic SARS-CoV-2 infected individuals in disseminating the infection (and sometimes to act as so-called ‘superspreaders') remains to be defined.

## LEARNING FROM THE SARS AND MERS OUTBREAKS

The propensity of SARS-CoV and MERS-CoV to generate large hospital outbreaks has been well established [27, 28]. The SARS epidemic in 2002-2003 emphasized that correct management of symptomatic cases within and outside hospital was crucial for containing the epidemic. In fact, the correct management of several imported cases of SARS into Vancouver (British Columbia, Canada), resulted in stopping secondary transmissions within the city. In contrast, inadequate application of infection control measures in Toronto (Ontario, Canada) and Taipei (Taiwan) led to significant hospital clusters and further spread [28]. The case of MERS is even more emblematic and enigmatic. MERS has no natural epidemic potential in the community and the transmission rate R0 in humans remains significantly below 1. Nevertheless, its potential to cause large and fatal hospital outbreaks is well established [29].

## COVID-19 OUTBREAKS IN ITALY AND SOUTH KOREA

An unexpected rise in the numbers of COVID-19 cases in northern Italy and South Korea in the third week of February 2020, indicated the global spread of the epidemic [30]. As of 25 February, 2020, 219 people have tested positive SARS-CoV-2 and towns in north of Italy were quarantined [30].

Preliminary information from Italy suggests that a large proportion of the primary clusters reported form Lombardy were actually linked to an emergency department where a symptomatic case was received on February 16^th^ (currently the whole hospital building is on quarantine). Similarly, the current COVID-19 spreading in South Korea has been largely fostered by several hospital outbreaks, the most recent of which occurred in a hospital in Cheongdo county, where COVID spread within the psychiatric unit [31]. In Lombardy, Veneto, Piedmont, Trentino-Alto Adige, and Friuli Venezia Giulia schools and public places like museums, monuments, libraries and tourist sites, were closed and public sporting events and other mass gatherings were banned. Patient zero for the Italian outbreak was sought after and spread by asymptomatic individuals considered.

## HOSPITAL OUTBREAKS OF NON-SARS-LIKE CoVs

The increased risk of spread of CoVs within healthcare services and of causing severe disease in susceptible individuals is not only restricted to SARS-CoV-1, MERS-CoV and SARS-CoV-2. Epidemiological analysis of the endemic non-SARS-like CoVs reveals a similar pattern. Severe infections with CoV-229E have been described in association with an outbreak in a neonatal intensive care unit (University Hospital, Brest, France) [32]. CoV-NL63 has recently been described to cause a fatal outbreak of SARI in a long-term care facility with a 27.7% infection rate and 1.5% lethality in Baton Rouge, Louisiana, USA [33]. Reports on hospital outbreaks of CoV-OC43 and CoV-HKU1 are scanty, and infections by these CoVs resulting in SARI are typically seen in frail subjects [34, 35]. The propensity for nosocomial spreading and the dominant role played by symptomatic patients in the spreading of the infection are supported by basic virology. CoVs, such as CoV-NL63, SARS-CoV-1 and SARS-CoV-2, use a specific receptor, angiotensin converting enzyme 2 (ACE2), a type I transmembrane metallocarboxypeptidase with homology to ACE, which is expressed in human airway epithelial cells, lung parenchyma, heart, lung, kidney and intestinal tract [36, 37]. Since ACE2 is primarily expressed in the lower respiratory tract, meaning that subjects exposed to index cases producing virus-containing aerosol (e.g. intubated patients or patients with high viral load and severe cough) are the most likely to develop primary (severe) pulmonary infection [17, 38–40].

## PATHOGENESIS, DISEASE SEVERITY AND EPIDEMIC SPREAD

Epidemiological studies suggest that SARS-CoV-2 has an intrinsic capacity to cause epidemic spread [41]. The current fatality rate for COVID-19 cases is about 3.4%, significantly less than SARS and MERS but potentially higher than those reported for endemic human non-SARS CoV infections [42]. During the first two months of the current outbreak, COVID-19 has spread rapidly throughout China and caused varying degrees of illness [19].

Several studies suggest that antibodies against non-SARS-CoVs are highly prevalent in the general population including in children, suggesting that most individuals have been infected by CoVs and have potentially developed a certain degree of (protective) immune response [43, 44]. There is no clear evidence on whether and how prior exposure to a strain of CoV can produce permanent immunity against the strain species or even cross-immunity for other CoV species [43]. Unlike other respiratory diseases that have a quadratic ("U"-shaped) lethality curve (killing infants and elderly, but sparing adults, presumably because adults have a higher chance to be immune against the infection), SARS-CoV-2 has a lethality that continuously rises with age (sparing children but mostly killing elderly). The severity and the clinical picture could be even related to the activation of exaggerated immune mechanism, causing uncontrolled inflammation as this has been suspected for SARS and MERS [45]. Hence, there is uncertainty on the impact of individual immune responses on the severity of SARS-like CoV infections.

The discrepancy between the severity of cases observed in China and those outside China could be a result of prior exposure community circulation of non-SARS-CoVs and their antigenic epitopes, leading to antibody-dependent enhancement (ADE) of SARS-CoV-2. ADE can elicit sustained inflammation, lymphopenia, and/or cytokine storm, which have been observed in severe cases and those who die. This might explain observed the geographic discrepancies of severe cases and lower mortality than SARS of MERS [46].

SARS-CoV-1 caused an important outbreak in 2002-2003, but it is currently though to be extinct [32]. This eradication of SARS-CoV-1 has been attributed to successful contention by public action, consisting in quarantining potentially affected persons and isolating affected areas. However, other factors might have played a role in the fall of the SARS epidemic leading to the eventual extinction of the virus. In particular, it is possibility that only a minority of the population was actually susceptible to develop severe illness (some HLA haplotypes reportedly conferred resistance to SARS-CoV-1, but these studies have not been confirmed or even disproven) [44, 47–50]. The hypothesis that SARS-CoV-1 (or other, antigenically similar CoV-1) have silently infected a significant proportion of the local population, inducing herd immunity needs to be confirmed. Indeed, immunity against the infection, or also patterns of semi-immunity (capacity of the immune system to avoid severe infection) may be due to cellular rather than humoral immune responses. Animal models suggest that the efficiency of T lymphocyte-mediated immune responses is pivotal for controlling MERS-CoV and SARS-CoV infections [51, 52]. Evidence in animals are confirmed by observational studies in humans suggesting that MERS-CoV-specific T-cell responses is a strong predictor of the clinical outcome in patients [53]. Of note, antibodies against MERS-CoV have been detected in a significant fraction of persons exposed to camels and dromedaries without any clinical evidence of prior MERS [54–56], suggesting that MERS-CoV can infect individuals in an asymptomatic fashion, yet induce signs of a (protective?) immune response.

## ROLES OF HUMORAL AND CELLULAR IMMUNE RESPONSES

There are currently no data on the specific role of either humoral or cellular immunity or innate immunity in patients recovering from COVID-19. Only highly specialized laboratories are able to conduct experiments to investigate immune responses against HLA class-I and class-II-restricted viral epitopes mediated by CD8+ and CD4+ T lymphocytes, respectively, to confirm the conjecture of a cellular (rather than humoral) immunity against SARS-CoV-2. Moreover, the T lymphocytes responsible for clinically relevant antiviral immune responses have high chances to be locally present in, or close to, respiratory epithelia but have comparatively low chances to be detectable in peripheral blood [57–59]. It is well possible that the exclusive detection of humoral immunity (antibodies against SARS-CoV-2) leads to an underestimation of the anti-SARS-CoV-2 immune responses. Thus, it is possible that the actual incidence of infections with SARS-CoV-2 is much higher than the observed number of clinically and serologically evident cases of COVID-19. In fact, a larger epidemic might be smoldering. This silent epidemic, made of mild and paucisymptomatic (usually flu-like) infections, could parallel the evident COVID-19 outbreaks that are detected when patients develop radiological or functional signs of pneumonitis and they are tested for SARS-CoV-2. This scenario may have two consequences. First, over the forthcoming months, symptomatic cases could haphazardly occur either as (apparently) sporadic cases or as epidemic clusters among frail subjects (e.g. as nosocomial outbreaks), driven by unrecognized occasional spreaders. Second, these occasional spreaders might accelerate the induction of immunity at the population level.

**Figure 1:**
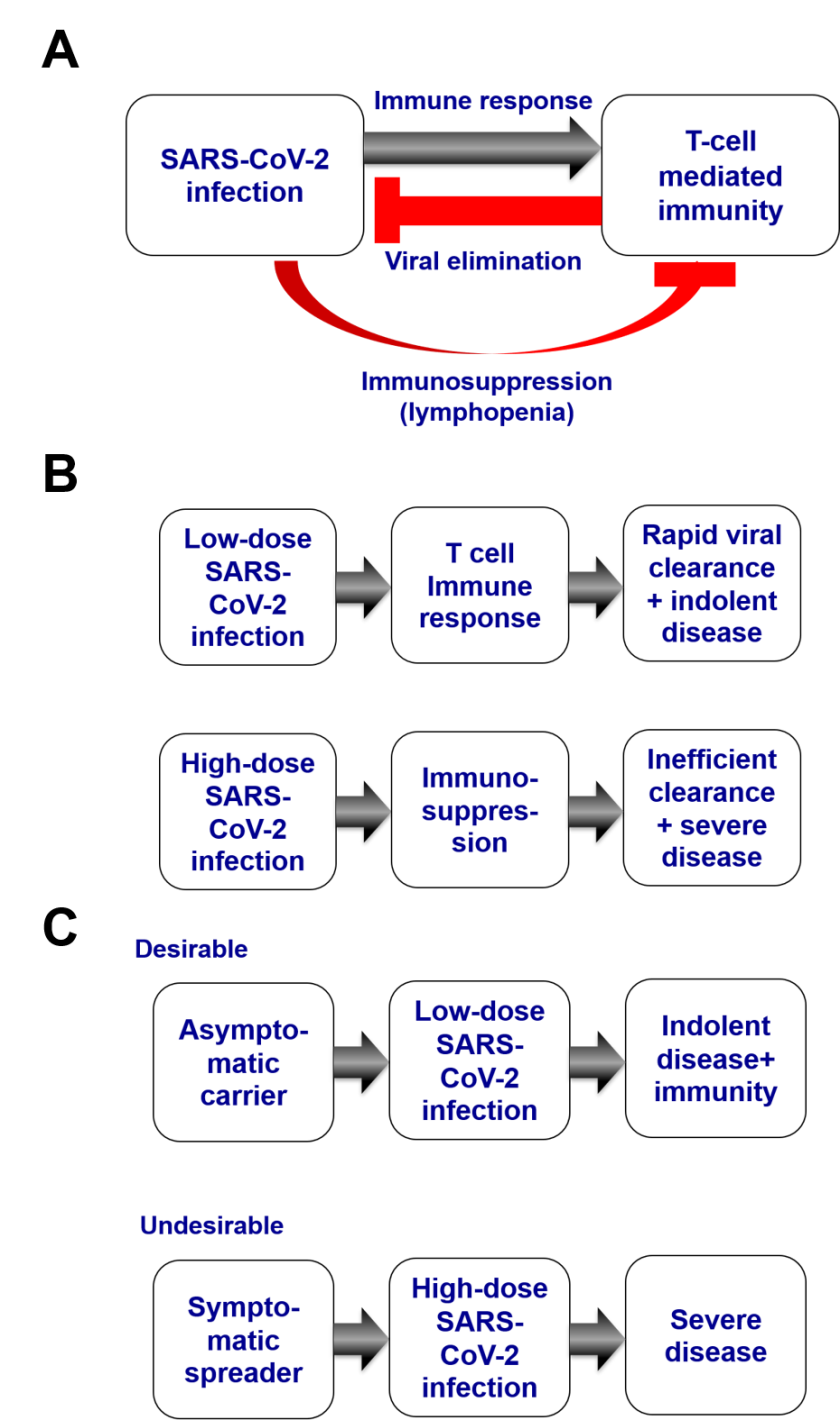
FIGURE 1: Proposed relationships between COVID-19 and anti-SARS-CoV-2 immune responses. **(A)** The precarious equilibrium between SARS-CoV-2 and the immune system. While the virus elicits an immune response leading to elimination of infected cells (and hence clearance of the infection), it tends to suppress the immune response. Therefore, the outcome of infection is determined by the kinetics of the immune response leading to viral elimination versus viral replication leading to immunosuppression. **(B)** Hypothetical effect of the initial contagion on the course of the infection caused by SARS-CoV-2. A low-level contagion would favor an efficient immune response and indolent infection, leading to immunity of the infected person. Transmission of a large number of viral particles would tend to cause multifocal respiratory infection leading to immunosuppression and severe illness and possibly death. **(C)** Possible modes of transmission of SARS-CoV-2. Indolent carriers would tend to transmit a low viral load to their contacts, leading to indolent disease and antiviral immune responses in immunocompetent individuals. This mode of transmission might be considered as desirable because it leads to population immunity if it attains a large fraction of the population. In contrast, symptomatic carriers might transmit larger amounts of viral particles with a higher probability of leading to severe disease.

By analogy to other CoVs, SARS-CoV-2 might induce a T-lymphocyte-mediated protective immune response. However, patients infected by SARS-CoV-2 that are hospitalized frequently manifest a lymphopenia, suggesting that cellular immune responses may be suppressed [60, 61]. In this context, it becomes plausible that, after infection by SARS-CoV-2, a sort of race decides the course of the events. Either a cellular immune response rapidly clears SARS-CoV-2 – in the best-case-scenario without any (or mild) clinical signs of infection – or the virus causes a state of immunosuppression that debilitates and sometimes overwhelms the host's defense **(Fig. 1A)**. In this context, the initial dose of the viral inoculum leading to infection may have a decisive impact on all subsequent events **(Fig. 1B)**. A small burden of SARS-CoV-2 should have a higher chance to stimulate a protective immune response than a high one, although additional factors like the fitness of the individual's immune system and prior exposure to other in part cross-reactive CoVs might influence the outcome of the race between viral replication and T-lymphocyte responses as well. Hence, it is possible, but remains to be demonstrated, that SARS-CoV-2 transmission from indolent or mildly symptomatic persons to naive individuals generally occurs at a relatively low viral load (lower than if the infection stems from severely affected patients), which then might have higher probabilities to induce immunity instead of severe and sometimes lethal infection **(Fig. 1C)**. That said, current evidence suggests that the most solid predictors of disease severity after infection with SARS-COV-2 are the patient's age and the concurrence of specific co-morbidities. In contrast, there is no proof (yet) that infection by a pauci-symptomatic person would result in a milder clinical course of immunocompetent neo-infected person.

## CHALLENGES FOR CONTROL OF THE EPIDEMIC

COVID-19 outbreak poses significant challenges for curtailing global spread and maintaining global health security. Implementation of collective infection control measures (e.g. social isolation, distancing or quarantine of entire communities) may be useful. Nonetheless, these measures should be implemented in a prudent fashion while considering their cost efficiency (e.g. for controlling small clusters of sporadic transmission). There is a real need to avoid an unmanageable epidemic wave that would saturate the capacity of health services. It is important to note that collective infection control measures can actually reduce the frequency of infection, though at the price of a prolongation of the epidemic period. Thus, in the absence of an effective vaccine and effective antiviral drugs, all infection control measures should be properly undertaken with the aim of modulating the trajectory of the epidemic so that the impact on global health is minimized and each epidemic wave does not exceed the healthcare system capabilities.

## TAKING FORWARD INTERVENTIONS TO CONTROL THE EPIDEMIC

The first pillar for interventions is to preserve the healthcare system. The implementation of infection control measures within hospitals is crucial to protect healthcare workers, maintain adequate work force levels and to prevent hospital outbreaks that eventually foster larger community epidemics.

Second, there is a growing need for providing advice on proper management of COVID-19 patients so that each individual can receive the most appropriate treatment. Currently, most of the SARS-CoV-2 infections need no therapy, and overtreatment of patients without current or future medical needs should be avoided.

Third, trust between people and institutions (at the local, national and international levels) must be maintained or reestablished so that local communities and individual subjects adhere to medical advice, for instance by respecting temporary individual restrictive measure (i.e. fiduciary isolation at home for mild-symptomatic cases of COVID-19).

Fourth, any antagonism between countries and their governments must be carefully avoided. The scientific community is global, by definition and for necessity. There is no individual solution for a globally spreading infection. Antagonism and lack of trust between countries will affect scientific collaboration and will retard or even jeopardize the control of SARS-CoV-2.

Fifth, research on effective prevention or treatment of COVID-19 must be accelerated. Yet unconfirmed reports indicated that inhibitors of SARS-CoV-2 replication including chloroquine are clinical efficient against declared SARS-CoV-2 infection [62–64]. If these findings are confirmed, chloroquine might be used to prophylactically treat vulnerable individuals (in particular the elderly and patients with existing medical problems) that have a high risk of viral exposure. Chloroquine has been used for decades for the prevention and treatment of malaria with minimal side effects and at a low cost, suggesting the practicability of such a measure.

Sixth, it is essential to control panic and to minimize the potential for social disruption that is typical of any global epidemic event. Again, exaggerated infection control measures may be pernicious as they increase frustration among the population, undermine the economy, and evoke a false feeling of safety.

Despite the fact that SARS-CoV-2 appears much less virulent than SARS-CoV-1 and MERS-CoV, it is associated with significant mortality among susceptible individuals with comorbidities. Moreover, the hype and scaremongering going viral on mass news and social media, predicting the dawn of a new fatal pandemic, are spurring global hysteria. Thus, the current COVID-19 epidemic is resulting in a social rather than a viral catastrophe. Whilst the future evolution of this epidemic remains unpredictable, classic public health strategies must follow rational patterns. The development of the response cannot be standardized as ‘one size fits all' but should be tailored based on the local evolution of the epidemic and the socio-economic settings involved. Indeed, the emergence of yet another global epidemic unveils the permanent challenge that infectious diseases represent for humankind and underscores the need for global cooperation and preparedness, even during inter-epidemic periods.

## Abbreviations:

ACE: – angiotensin converting enzyme;
ADE: – antibody-dependent enhancement;
ARDS: – acute respiratory distress syndrome;
CoV: – coronavirus;
COVID-19: – coronavirus disease 2019;
HCoV: – human coronavirus;
MERS: – Middle-East respiratory syndrome;
SARI: – severe acute respiratory illness;
SARS: – severe acute respiratory syndrome.
